# Emergence of Ciprofloxacin-Resistant *Salmonella enterica* Serovar Typhi in Italy

**DOI:** 10.1371/journal.pone.0132065

**Published:** 2015-06-29

**Authors:** Aurora García-Fernández, Silvia Gallina, Slawomir Owczarek, Anna Maria Dionisi, Ildo Benedetti, Lucia Decastelli, Ida Luzzi

**Affiliations:** 1 Department of Infectious, Parasitic and Immune-Mediated Diseases, Istituto Superiore di Sanità, Rome, Italy; 2 Regional Reference Centre for Salmonella serotyping, Istituto Zooprofilattico Sperimentale del Piemonte, Liguria e Valle d'Aosta, Turin, Italy; 3 Food Hygiene and Safety Department, Istituto Zooprofilattico Sperimentale del Piemonte, Liguria e Valle d'Aosta, Turin, Italy; Institut National de la Recherche Agronomique, FRANCE

## Abstract

In developed countries, typhoid fever is often associated with persons who travel to endemic areas or immigrate from them. Typhoid fever is a systemic infection caused by *Salmonella enterica* serovar Typhi. Because of the emergence of antimicrobial resistance to standard first-line drugs, fluoroquinolones are the drugs of choice. Resistance to ciprofloxacin by this *Salmonella* serovar represents an emerging public health issue. Two *S*. *enterica* ser. Typhi strains resistant to ciprofloxacin (CIP) were reported to the Italian surveillance system for foodborne and waterborne diseases (EnterNet-Italia) in 2013. The strains were isolated from two Italian tourists upon their arrival from India. A retrospective analysis of 17 other *S*. *enterica* ser. Typhi strains isolated in Italy during 2011–2013 was performed to determine their resistance to CIP. For this purpose, we assayed for susceptibility to antimicrobial agents and conducted PCR and nucleotide sequence analyses. Moreover, all strains were typed using pulsed-field gel electrophoresis to evaluate possible clonal relationships. Sixty-eight percent of the *S*. *enterica* ser. Typhi strains were resistant to CIP (MICs, 0.125–16 mg/L), and all isolates were negative for determinants of plasmid-mediated quinolone resistance. Analysis of sequences encoding DNA gyrase and topoisomerase IV subunits revealed mutations in *gyrA*, *gyrB*, and *parC*. Thirteen different clonal groups were detected, and the two CIP-resistant strains isolated from the individuals who visited India exhibited the same PFGE pattern. Because of these findings, the emergence of CIP-resistant *S*. *enterica* ser. Typhi isolates in Italy deserves attention, and monitoring antibiotic susceptibility is important for efficiently managing cases of typhoid fever.

## Introduction

Typhoid fever is a systemic infection caused mainly by *Salmonella enterica* serovar Typhi. This disease is a major burden in developing countries and accounts for more than 20 million cases and 200,000–600,000 deaths annually worldwide. Approximately 90% of deaths occur in Asia (http://www.who.int/immunization/sage/SAGE_Background_paper_typhoid_newVaccines.pdf). With the widespread emergence and spread of *S*. *enterica* ser. Typhi strains that are resistant to chloramphenicol, ampicillin, and trimethoprim, ciprofloxacin (CIP) is now the first-line drug of choice for treating typhoid fever. However, the frequency of strains resistant to nalidixic acid (NAL) and CIP has increased during the last 10 years [1]. *S*. *enterica* ser. Typhi strains isolated from Asia, Latin America, and Africa share this pattern of drug resistance [1]. Clinical evidence indicates a poor response to systemic infections caused by *S*. *enterica* ser. Typhi strains that are resistant to CIP, e.g. the minimum inhibitory concentration (MIC) > 0.06 mg/L (EUCAST v.5.0, 2015) [2, 3].

Quinolone resistance among *Salmonella* spp. is frequently associated with mutations in the genes encoding DNA gyrase (*gyrA* and *gyrB*) or topoisomerase (*parC* and *parE*) that reside in the quinolone resistance-determining region (QRDR) [4, 5]. Plasmid genes that mediate quinolone resistance (PMQR), including *qnr* (*qnrA*, *qnrB*, *qnrS*, *qnrC*, *and qnrD*), *qepA*, *oqxAB*, and *aac(6')-Ib-cr*, are present in *Salmonella* species that are resistant to quinolones but only *qnrB*, *qnrS*, and *aac(6')-Ib-cr* genes have been described in *S*. *enterica* ser. Typhi [6–8]. The quinolone resistance of *Salmonella* spp. has also been associated with decreased permeability to quinolones and overexpression of efflux pumps [9].

Typhoid fever is a rare disease in Italy, and approximately 20 confirmed cases are reported yearly to the National Surveillance System for Foodborne and Waterborne Diseases, which is coordinated by the *Istituto Superiore di Sanità* (EnterNet Italia, www.iss.it/ente). Typhoid fever occurs mainly in individuals who travel to endemic areas as well as in immigrants from these regions. Initial treatment of enteric fever typically employs empirical antimicrobial therapy before the results of drug-sensitivity tests are available. To prevent possible treatment failures, it is important for healthcare providers to possess knowledge of the antibiotic resistance of *S*. *enterica* ser. Typhi [10]. In particular, monitoring the antibiotic susceptibility spectrum of this serovar in Italy is important for efficiently managing patients with typhoid fever who contracted the disease in endemic areas and for preventing the spread of the disease.

Therefore, the aim of the present study was to characterize *S*. *enterica* ser. Typhi strains resistant to CIP that were isolated in Italy from 2011 to 2013. For this purpose, we determined the antimicrobial resistance patterns of these strains, characterized the relationships among strains by using pulse-field gel electrophoresis (PFGE), and performed nucleotide sequence analysis to identify the mutations responsible for drug resistance.

## Material and Methods

### Bacterial isolates

Over a 3-year period (from 2011 to 2013), only 19 of the 106 *S*. *enterica* ser. Typhi strains reported to the Italian surveillance system for enteric pathogens (EnterNet Italia) were received at our Institution. All isolates were confirmed as *S*. *enterica* ser. Typhi by using the serotyping Kauffmann–White scheme [11]. The data reported in this study were extracted from an anonymous national surveillance network for human gastrointestinal infections (EnterNet Italia). Because no confidential patient information was used, an ethics statement is not applicable, and Institutional Review Board approval or informed consent of patients was not required.

### Assays of antimicrobial susceptibility

Antibiotic susceptibility tests were performed using the disk diffusion method with antimicrobial discs (Becton Dickinson, MD 21152–0999, USA), and the antibiotic concentrations (μg) were as follows: nalidixic acid (NAL, 30), ampicillin (A, 10), cefotaxime (CTX, 5), ceftazidime (CAZ, 10), amoxicillin/clavulanic acid 2:1 (AMC, 30), meropenem (MEM, 10), chloramphenicol (C, 30), gentamicin (G, 10), kanamycin (K, 30), streptomycin (S, 10), sulfonamides (Su, 0.25), tetracycline (T, 30), trimethoprim (TMP, 5), and trimethoprim–sulfamethoxazole (SXT, 1.25/23.75). The reference strain *Escherichia coli* ATCC 25922 was used as a control for each experiment. The MIC of CIP was determined using Etest strips (AB Biodisk, S-169 56 Solna, Sweden). The data were interpreted using the EUCAST guidelines (http://www.eucast.org/clinical_breakpoints/v.5.0) that define CIP resistance and sensitivity as MICs > 0.06 mg/L and ≤ 0.06 mg/L, respectively.

### Identification of genetic determinants of quinolone resistance

The chromosomal quinolone resistance-determining regions (QRDRs) *gyrA*, *gyrB*, *parC*, and *parE* [5] and plasmid-mediated quinolone resistance regions (PMQRs) *qnrA*, *qnrB*, *qnrC*, *qnrD*, *qnrS*, *aac(6’)-Ib-cr*, *qepA*, and *oqxAB* were amplified using PCR [12–14] and the amplicons were subjected to nucleotide sequence analysis.

### PFGE

**Fig 1:**
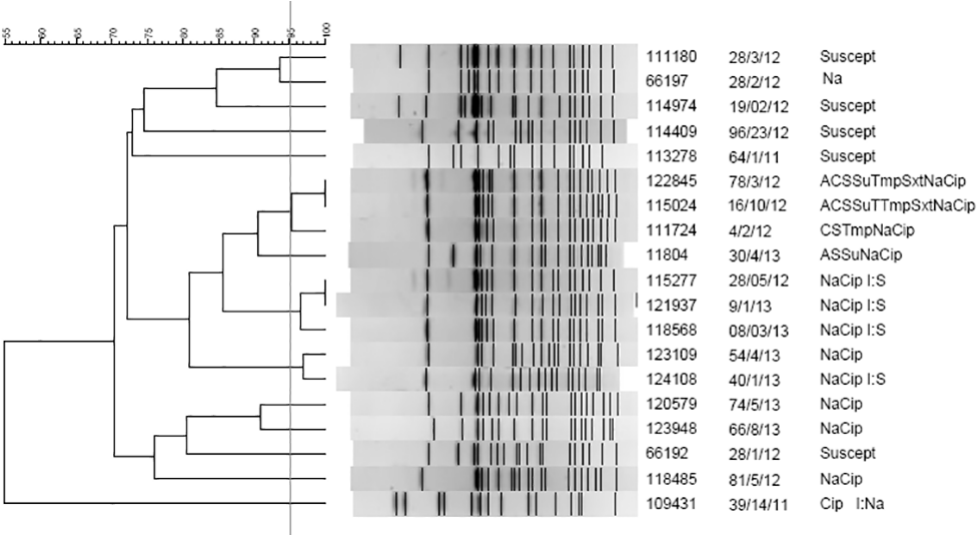
*S*. *enterica* ser. Typhi PFGE and Dendrogram.

Clonal relationships among the strains were assessed using PFGE according to the PulseNet protocol [15]. Genomic DNA was digested with XbaI (New England Biolabs, Ipswich, MA, USA), and *Salmonella enterica* serovar Braenderup H9812 DNA was used as the molecular size marker. Dendrogram and cluster analyses were performed using algorithms included in the BioNumerics software package v.6.6 (Applied Maths, Sint-Martens-Latem, Belgium). The percentage similarity between different chromosomal fingerprints was scored using the Dice coefficient. The unweighted pair group method with arithmetic means (UPGMA) with a 1.00% tolerance limit and 1.00% optimization was used to generate the dendrogram. A clonal relationship among strains was defined as a coefficient of similarity ≥ 95%. The clonal groups were named alphabetically from A to N (Fig 1).

## Results

### *S*. *enterica* ser. Typhi strains

Nineteen *S*. *enterica* ser. Typhi strains isolated in 2011 (n = 6), 2012 (n = 7), and 2013 (n = 6) were deposited in the collection of the *Istituto Superiore di Sanità*. The strains were isolated from blood (n = 7), urine (n = 1), feces (n = 7), or an unknown source (n = 4). The ages of patients ranged from 0 to 47 years (seven males, six females, and six unknown). The country of birth was known for 15 of the 19 patients, and four of the 19 patients traveled before presenting with typhoid fever (see Table 1). All the patients were residing in Italy at the time of the study. No other epidemiologically relevant information such as recent travels to endemic areas or recent immigration to Italy was available for 15 of these patients.

**Table 1:** *S*. *enterica* ser. Typhi Strains Isolated in Italy from 2011 to 2013.

Strain	Region	Date	Sample	Gender	Age	Origin	Travel	Resistance pattern	CIP MIC(mg/L)	GyrA	GyrB	ParC	ParE	PMQR	PFGE
**28/1/12**	CAMPANIA	2011	unk	unk	unk	unk	unk	Suscept	0.008	wt	wt	wt	wt	neg	L
**28/4/12**	CAMPANIA	2012	feces	unk	unk	Italian	unk	Suscept	0.008	wt	wt	wt	wt	neg	C
**28/3/12**	CAMPANIA	2011	feces	unk		Italian	unk	Suscept	0.008	wt	wt	wt	wt	neg	A
**96/23/12**	CAMPANIA	2012	unk	unk	unk	unk	unk	Suscept	0.015	wt	wt	wt	wt	neg	D
**64/1/11**	FRIULI V.G.	2011	urine	M	20	African	unk	Suscept	0.015	wt	wt	wt	wt	neg	E
**28/2/12**	CAMPANIA	2011	unk	unk	unk	unk	unk	Na	0.032	Asp82Asn	wt	wt	wt	neg	B
**39/14/11**	MARCHE	2011	blood	M	0	Indian	unk	Cip I:Na	0.125	wt	wt	Thr57Ser	wt	neg	N
**81/5/12**	LAZIO	2012	feces	M	14	Bangladesh	unk	NaCip	0.15	Ser83Phe	Gly435Ala	wt	wt	neg	M
**66/8/13**	LAZIO	2013	blood	F	13	Bangladesh	unk	NaCip	0.19	Asp87Asn	Gly435Glu	wt	wt	neg	K
**30/4/13**	PIEMONTE	2013	unk	unk	unk	unk	unk	ASSuNaCip	0.25	Ser83Phe	wt	wt	wt	neg	G
**4/2/12**	TRENTO	2011	blood	F	16	Bangladesh	unk	CSTmpNaCip	0.25	Ser83Phe	wt	wt	wt	neg	F
**78/3/12**	PIEMONTE	2012	feces	F	34	Bangladesh	Bangladesh	ACSSuTmpSxtNaCip	0.25	Ser83Phe	Gly435Glu	wt	wt	neg	F
**16/10/12**	LAZIO	2012	feces	M	unk	Bangladesh	unk	ACSSuTTmpSxtNaCip	0.25	Ser83Phe	wt	wt	wt	neg	F
**28/5/12**	CAMPANIA	2012	blood	F	33	Indian	unk	NaCip I:S	0.25	Ser83Phe	wt	wt	wt	neg	H
**8/3/13**	TRENTO	2012	blood	M	3	Bangladesh	unk	NaCip I:S	0.25	Ser83Phe	wt	wt	wt	neg	H
**9/1/13**	CAMPANIA	2013	blood	M	29	Italian	India	NaCip I:S	0.38	Ser83Tyr	wt	wt	Ser493Phe	neg	H
**74/5/13**	MARCHE	2013	feces	M	32	Indian	unk	NaCip	0.5	Ser83Phe	Gly435Val	wt	wt	neg	J
**40/1/13**	PIEMONTE	2013	feces	F	40	Italian	India	NaCip I:S	12	Ser83Phe,Asp87Asn	Gly435Glu	Ser80Ile	wt	neg	I
**54/4/13**	PIEMONTE	2013	blood	F	47	Italian	India	NaCip	16	Ser83Phe,Asp87Asn	wt	Ser80Ile	wt	neg	I

I: Intermediate susceptibility; wt: wild-type; unk: unknown; suscept: susceptible to all the antimicrobials tested.

### Antimicrobial susceptibility and analysis of QRDRs and PMQRs

Sixty-eight percent (13/19) of the *S*. *enterica* ser. Typhi strains were resistant to CIP. All isolates were susceptible to third-generation cephalosporins. PMQR genes were not detected, and five isolates were susceptible to all the antibiotics tested (MICs for NAL and CIP MIC ranged between 0.008–0.015 mg/L) (Table 1). Further, mutations in the QRDR region were not detected. One strain resistant only to NAL harbored the Asp82Asn GyrA mutation (CIP MIC = 0.032 mg/L). One CIP-resistant strain (MIC = 0.125 mg/L) with intermediate susceptibility to NAL had one ParC mutation (Thr57Ser). Twelve NAL–CIP-resistant strains (MICs 0.15–16 mg/L) harbored mutations in GyrA, GyrB, ParC, and ParE that conferred different levels of resistance to CIP. The two strains isolated from two Italian patients who traveled together to India were highly resistant to CIP (MICs = 12 and 16 mg/L). The GyrA mutations Ser83Phe-Asp87Asn and ParC Ser80Ile were detected in the isolates from each person. The strain with CIP MIC = 12 mg/L harbored the GyrB Gly435Glu point mutation (Table 1). The NAL-CIP resistant strains with CIP MIC values of 0.15 to 0.5 mg/L harbored single mutations in GyrA (Ser83Phe, Ser83Val, or Asp87Asn), some of which were associated with single mutations of GyrB (Gly435Ala, Gly435Glu, or Gly435Val) or a single ParE mutation (Ser493Phe). Four of these NAL–CIP resistant strains were multidrug-resistant (MDR) (Table 1).

### Clonal relationships

PFGE analysis revealed 14 different restriction patterns among the 19 isolates. Only three clonal groups were established with a maximum of three isolates (Fig 1). Three of the four MDR strains were included in the same group (F) and were isolated from three natives of Bangladesh (one of whom had traveled to Bangladesh before presenting with illness) at three regions from Italy in different years. Clonal group H comprised three strains that were resistant to NAL and CIP, with intermediate susceptibility to streptomycin. These strains were isolated from one native of Bangladesh, one native of India, and one Italian who had traveled to India. Clonal group I consisted of two CIP-resistant strains isolated from the two Italian patients who had traveled together to India.

## Discussion

Certain diseases that are not endemic or completely eradicated can reoccur sporadically. Because of the increase in the immigrant population in Italy and travel by people from countries with endemic typhoid fever, *S*. *enterica* ser. Typhi is occasionally isolated in Italy. From 2011 through 2013, the Italian surveillance system for enteric pathogens (EnterNet Italia), reported the isolation of 193 106 *S*. *enterica* ser. Typhi strains isolated from blood, urine, feces, or combinations of these sources. Only 19 of these strains were received at our institution. Ten strains were isolated from three natives of India, six were from Bangladesh, one was from Africa, and five were isolated from Italian patients. Three of the five Italian patients had traveled to India and whether the other two had traveled is unknown. No clonal relationships were detected that indicated the possibility of a typhoid fever outbreak in Italy.

While uncommon in Italy, typhoid fever is highly endemic in developing countries in Africa, Asia (especially Southeast Asia and the Indian subcontinent), and Central and South America. PFGE typing of all the *S*. *enterica* ser. Typhi strains isolated in the 2011–2013 period produced only three clonal groups. Identical clones were associated with natives of different endemic countries (India and Bangladesh) (clone F), suggesting the distribution of the same clone. Clone H was composed of three identical clones from three unrelated patients from Bangladesh, which indicates that this particular clone could be widespread in Bangladesh. Clonal group I included two CIP-resistant strains isolated from two Italian patients who had traveled together to India.

With the global emergence of *S*. *enterica* ser. Typhi isolates that are resistant to chloramphenicol, ampicillin, and trimethoprim-sulfamethoxazole, as well as others, CIP is the first-line drug for treating typhoid fever [16]. After the introduction of fluoroquinolones as therapy for typhoid fever, treatment failure was reported using CIP [17]. The widespread use of fluoroquinolones is associated with the dissemination of CIP-resistant strains [18]. Here, we show that five (26%) of the *S*. *enterica* ser. Typhi isolates were susceptible to all the antimicrobials tested, 12 (63%) were resistant to NAL and CIP, and only four were MDR strains.

With the recent reduction of the MIC breakpoints for resistance to CIP recommended by the EUCAST (http://www.eucast.org/clinical_breakpoints/v.5.0), the NAL–CIP-resistant strains predominated in the present study. A decrease in the number of MDR strains and an increase in the number of fluoroquinolone-resistant strains of *S*. *enterica* ser. Typhi isolated from patients residing in the Indian subcontinent and certain regions of Southeast Asia were observed [19–21]. Therefore, it was proposed that drugs other than fluoroquinolones such as oral azithromycin or intravenous ceftriaxone might serve as an effective initial therapy for patients with enteric fever from those countries [19, 22]. Fortunately, resistance to third-generation cephalosporins is rarely encountered [19]. Moreover, the World Health Organization recommends the programmatic use of licensed vaccines (Vi and Ty21a) for controlling endemic disease in specific areas where the burden of disease is great and rates of antibiotic resistance are high (http://www.who.int/immunization/diseases/typhoid/en/).

Fluoroquinolones target DNA gyrase and topoisomerase IV, which comprise subunits encoded by *gyrA*–*gyrB* and *parC*–*parE*, respectively. Single point mutations in *gyrA* confer resistance to NAL, and the cumulative effects of mutations in *gyr* and *par* may confer resistance to CIP [23]. In the present study, the NAL-CIP-resistant strains harbored different mutations in GyrA, four in GyrB, and one in ParE (Table 1). A single amino acid residue substitution in *S*. *enterica* ser. Typhi GyrA conferred resistance to NAL and cinoxacin. In contrast, resistance to CIP and other fluoroquinolones requires two substitutions in GyrA and one in ParC [24–26]. In the strains tested here, according to the new EUCAST CIP breakpoints, we showed that a single GyrA mutation conferred resistance to CIP. Further, these GyrA mutations were frequently detected in other studies and include Ser83Phe, which is one of the most frequent mutations [27–28]; ParC mutations were also reported [21].

The two NAL–CIP-resistant strains (MICs of 12 and 16 mg/L) harbor three point mutations as follows: GyrA (Ser83Phe, Asp87Asn) and ParC (Ser80Ile). The high levels of resistance observed for the triple point mutants indicate that the amino acid residue substitutions protect GyrA and ParC from relatively high concentrations of fluoroquinolones [24]. The identical triple GyrA–ParC mutations identified here in two NAL–CIP resistant strains (CIP MIC = 8 mg/L) were detected in one *S*. *enterica* ser. Typhi isolate from Nepal in 2012 [25] and in two *S*. *enterica* ser. Typhi strains (CIP MIC > 32 mg/L) from Taiwan in 2011 [26]. Further, two NAL-CIP resistant strains (CIP MICs 64 and 6 mg/L) with the same triple mutation were isolated in India during 2005–2009, although these strains expressed an efflux pump that contributed to their fluoroquinolone resistance [29].

There is a re-emergence of susceptibility to first-line antibiotics and the decline of MDR *S*. *enterica* ser. Typhi strains. Our strains were mostly resistant to NAL and CIP due to mutations in the QRDR locus and were susceptible to third-generation cephalosporins. Because this is the first report of the isolation of *S*. *enterica* ser. Typhi strains resistant to NAL and CIP in Italy, prudent use of quinolones as empirical therapy for typhoid fever is required to prevent emergence of resistant strains that cause treatment failures. Good surveillance is required to target CIP-resistant *S*. *enterica* ser. Typhi that may cause treatment failure. Moreover, improving vaccination of the inhabitants of endemic areas and publicizing the *S*. *enterica* ser. Typhi immunization program to people who plan to travel to these regions may serve as effective measures to prevent the dissemination of enteric fever.
